# Histone methyltransferase SUV39H2 regulates cell growth and chemosensitivity in glioma via regulation of hedgehog signaling

**DOI:** 10.1186/s12935-019-0982-z

**Published:** 2019-10-16

**Authors:** Ran Wang, Lilin Cheng, Xi Yang, Xin Chen, Yifeng Miao, Yongming Qiu, Zhiyi Zhou

**Affiliations:** Department of Neurosurgery, South Campus, Renji Hospital, School of Medicine, Shanghai Jiao Tong University, Shanghai, China

**Keywords:** SUV39H2, Glioma, Cancer stem cell, Hh signaling, HHIP

## Abstract

**Background:**

Malignant glioma is one of the essentially incurable tumors with chemoresistance and tumor recurrence. As a histone methyltransferase, SUV39H2 can trimethylate H3K9. SUV39H2 is highly expressed in many types of human tumors, while the function of SUV39H2 in the development and progression of glioma has never been elucidated.

**Methods:**

RT-qPCR and IHC were used to test SUV39H2 levels in glioma tissues and paired normal tissues. The clinical relevance of SUV39H2 in glioma was analyzed in a public database. Colony formation assays, CCK-8 assays, and flow cytometry were conducted to explore the role of SUV39H2 in the growth of glioma cells in vitro. A cell line-derived xenograft model was applied to explore SUV39H2’s role in U251 cell proliferation in vivo. Sphere formation assays, RT-qPCR, flow cytometry, and IF were conducted to illustrate the role of SUV39H2 in the stemness and chemosensitivity of glioma. Luciferase reporter assays and WB were applied to determine the function of SUV39H2 in Hh signaling.

**Results:**

SUV39H2 was highly expressed in glioma tissues relative to normal tissues. SUV39H2 knockdown inhibited cell proliferation and stemness and promoted the chemosensitivity of glioma cells in vitro. In addition, SUV39H2 knockdown also significantly inhibited glioma cell growth in vivo. Moreover, we further uncovered that SUV39H2 regulated hedgehog signaling by repressing HHIP expression.

**Conclusions:**

Our findings delineate the role of SUV39H2 in glioma cell growth and chemosensitivity as a pivotal regulator of the hedgehog signaling pathway and may support SUV39H2 as a potential target for diagnosis and therapy in glioma management.

## Background

Malignant gliomas, one of the essentially incurable tumors, account for up to 80% of all primary brain tumors [1]. There are 3 categories of gliomas: astrocytoma, oligodendroglioma and glioblastoma (GBM) [2]. Based on histopathological and genetic parameters, malignant gliomas can be classified into four grades (low-grade: astrocytomas or oligodendrogliomas; high-grade: astrocytomas or GBM) [3]. Among these, GBM is the most common and most malignant brain tumor with the worst prognosis [4]. For newly diagnosed primary GBM, even after the gold standard treatment (successful surgical resection, fractionated radiation, and chemotherapy with temozolomide (TMZ)) [5, 6], the outcome still remains poor because of tumor recurrence. The median survival time negligibly increases from 12.1 to 14.6 months [7]. The depressing prognosis of GBM is caused by chemoresistance to TMZ and other drugs. TMZ, an alkylating cytostatic, is the frontline chemotherapeutic for GBM that acts by inducing DNA damage and leads to cell death [8]. Thus, many studies are needed to discover new targets and to elucidate the underlying mechanism of therapy resistance and tumor recurrence.

Glioma stem-like cells (GSCs) are an aggressive subset with stem cell features within glioma cells and have been implicated in tumor recurrence and drug resistance. GSCs have the capacity to self-renew, are pluripotent, and induce tumorigenesis [9–12]. It has been reported that the hedgehog (Hh), Wnt, and Notch pathways play important roles in glioma progression [13–15]. In particular, the Hh pathway is a potentially effective target for treating GBM because it is indispensable for the tumorigenesis of GSC [14]. Hedgehog interacting protein (HHIP) negatively regulates the Hh pathway by binding to the hedgehog protein and acts as a tumor suppressor [16]. However, the regulatory mechanism of HHIP in glioma is still unclear.

Recent studies have provided evidence that the interplay of genetic and epigenetic mechanisms accounts for the pathogenesis and progression of glioma [17]. Among the epigenetic changes contributing to the development of gliomas, histone methylation is one of the most investigated. SUV39H2, a suppressor of variegation 3–9 homolog 2, specifically catalyzes lysine 9 of histone 3 (H3K9) trimethylation [18], which generally leads to gene silencing. Additionally, SUV39H2 was reported to be highly expressed in a number of tumors, including bladder cancer [19], hepatoma [20], acute lymphoblastic leukemia [21], lung adenocarcinoma [22], and nasopharyngeal carcinoma [23]. Nevertheless, the function of SUV39H2 in glioma progression remains unclear and needs to be further elucidated. The major aim of this study was to ascertain the function of SUV39H2 in tumorigenesis and explore its underlying mechanisms in glioma.

Here, we report that SUV39H2 is highly expressed in human glioma tissues. SUV39H2 knockdown inhibits the growth of glioma cells in vitro and in vivo. In addition, SUV39H2 knockdown enhances stem cell properties and TMZ sensitivity in glioma cells by regulating the Hh pathway. Therefore, our results emphasize that the inhibition of SUV39H2 expression can be a target for glioma therapy.

## Materials and methods

### Human specimen analysis

Glioma and paracancerous tissue specimens (26 patients) were acquired from Renji Hospital (Shanghai, China). Patient specimens were obtained from glioma patients (without preoperative chemotherapy or radiation) after surgical resection. The grading of gliomas was based on the latest World Health organization (WHO) classification. The study protocol was approved by the Ethics Committee of Renji Hospital (Shanghai, China).

### Cell lines, plasmids, and cell transduction

Cell lines were purchased from the ATCC. We purchased RBP-Jκ, GLI, and TCF/LEF1 luciferase reporters from Shanghai Genomeditech. DMEM (containing 10% fetal bovine serum) was used to culture cells. Lipofectamine 3000 (Invitrogen) was used to transfect cell lines according to the manufacturer’s instructions. We cloned a gene-specific shRNA into the pLVX-shRNA1 plasmid (Clontech) to knockdown genes and a scrambled shRNA sequence as a control. We used the 3-plasmid system to package lentiviruses using psPAX2 and pMD2G. After transduction with the lentivirus and screening with puromycin, stable U251 and U87 cell lines were obtained. The sequences of the shRNAs are listed in Additional file 1: Table S1.

### Extraction of RNA and real-time reverse transcription polymerase chain reaction (RT-qPCR)

RNAiso Plus Reagent (TaKaRa) was applied to extract total cellular RNA, and the PrimeScript RT Reagent Kit (Perfect Real Time, TaKaRa) was applied to reverse transcribe RNA (1.5 μg) as instructed by the manufacturer. We performed RT-qPCR on a 7500 Fast Real-Time PCR System (Applied Biosystems, Carlsbad, CA). GAPDH was used for normalization. The sequences of the primers are listed in Additional file 1: Table S2.

### Western blot (WB) analysis

Protein was harvested using lysis buffer, and a BCA assay was applied to determine the protein concentration. Next, we separated 20 μg of protein using sodium dodecyl sulfate–polyacrylamide gel electrophoresis (SDS-PAGE) and transferred protein to a polyvinylidene difluoride (PVDF) membrane (Millipore, MA). After blocking, membranes were incubated with primary antibodies as indicated and with horseradish peroxidase (HRP)-conjugated secondary antibodies. Membranes were developed using a chemiluminescent substrate (Beyotime, China). The antibodies used are listed in Additional file 1: Table S3.

### Immunohistochemistry (IHC)

Glioma tissues were fixed with formalin, embedded in paraffin, and sliced into 5-μm-thick sections. Then, the samples underwent a strict process of deparaffinization, rehydration, antigen retrieval, and endogenous peroxidase inhibition. Next, sections were immunoblotted with an SUV39H2 antibody overnight (4 °C). We then washed the membranes and incubated them with the appropriate secondary antibody. As a chromogen, diaminobenzidine (DAKO) solution was applied to visualize samples. At last, nuclei were stained with hematoxylin. A microscope (Leica DM 4000B) was used to acquire images. The antibodies used are listed in Additional file 1: Table S3.

### Cell Counting Kit-8 (CCK-8) assay

We used the Cell Counting Kit-8 assay (MedChemExpress, USA) to study glioma cell viability as guided by the manufacturer. We seeded a total of 2000 cells/well into a 96-well plate. After the indicated times, we added CCK-8 solution (10 µl) and then used a microplate reader to measure the absorbance at A450.

### Colony formation assay

We seeded 1000 cells into 6 cm plates for 7 days. The colonies were then dyed with crystal violet staining solution (Sangon Biotech, China) after fixation with 4% paraformaldehyde. At last, we counted the number of colonies.

### Flow cytometry

Stable SUV39H2-knockdown and control cells were cultured in 6-well plates, harvested at the indicated times, and subjected to flow cytometry. A Cell-Light EdU Kit (C10338: Ruibo) was used to determine cell cycle analysis. To examine apoptotic cells, we stained cells with annexin-V and 7AAD (BD Biosciences). Data were processed using FlowJo 7.6 (Tree Star, Inc.) and Kaluza (Beckman Coulter).

### Tumorigenesis assay in vivo

We injected 2 × 10^6^ lentiviral-transduced glioma cells (suspended in 200 μl PBS) subcutaneously into the right flanks of nude mice. We recorded tumor growth weekly and used the formula (tumor length × tumor width × tumor width*1/2) to calculate the tumor volume. After euthanasia, we imaged and weighed the tumors. We handled the experimental animals according to the Guide for the Care and Use of Laboratory Animals.

### In vivo tumor initiation assay

For in vivo limiting dilution studies, glioma cells, at dilutions of 1 × 10^5^, 1 × 10^4^, and 1 × 10^3^ cells in 200 μl PBS, were injected subcutaneously into the right flanks of nude mice. Tumor incidence was determined at the indicated time points by calculating the number of tumors. We handled the experimental animals according to the Guide for the Care and Use of Laboratory Animals.

### Sphere formation assay

We seeded 2000 cells/well into a 6 cm ultralow attachment plate (Corning). We used DMEM F12 medium (Gibco) supplemented with human EGF (20 ng/ml, Peprotech) and human bFGF (20 ng/ml, Peprotech) to culture cells for 10 days. The number of spheres (> 100 μm) was counted under a Nikon light microscope.

### Immunofluorescence (IF)

We fixed samples with cooled acetone for 10 min and permeabilized samples with PBS containing 0.3% Triton X for 5 min. Then, we blocked cells using 10% BSA buffer (2 h, 25 °C). Samples were incubated with a γH2A.X antibody at 4 °C overnight. Then, we washed samples thrice thoroughly using PBS. We then incubated samples in the dark using fluorochrome-conjugated secondary antibodies (1 h, 25 °C). After washing thrice, the samples were stained with DAPI. The samples were mounted and visualized using a fluorescence microscope. The antibodies used are listed in Additional file 1: Table S3.

### Luciferase reporter assay

We cotransfected glioma cells with the indicated luciferase reporters and Renilla luciferase. The luciferase kit (Promega, E1910) was applied to determine the activities of Renilla luciferase and firefly luciferase as instructed by the manufacturer. The relative activity of firefly luciferase was quantified after normalization to the activity of Renilla luciferase.

### Statistical analysis

We conducted experiments at least thrice. Data are presented as the mean ± SEM. We analyzed differences between groups using Student’s *t* test. We analyzed the correlation between SUV39H2 and HHIP by Pearson’s correlation test. We analyzed the Kaplan–Meier survival curve comparison by the log-rank test. We used SPSS to analyze the data. p < 0.05 was considered statistically significant.

## Results

### SUV39H2 is upregulated in human glioma cells

**Fig. 1:**
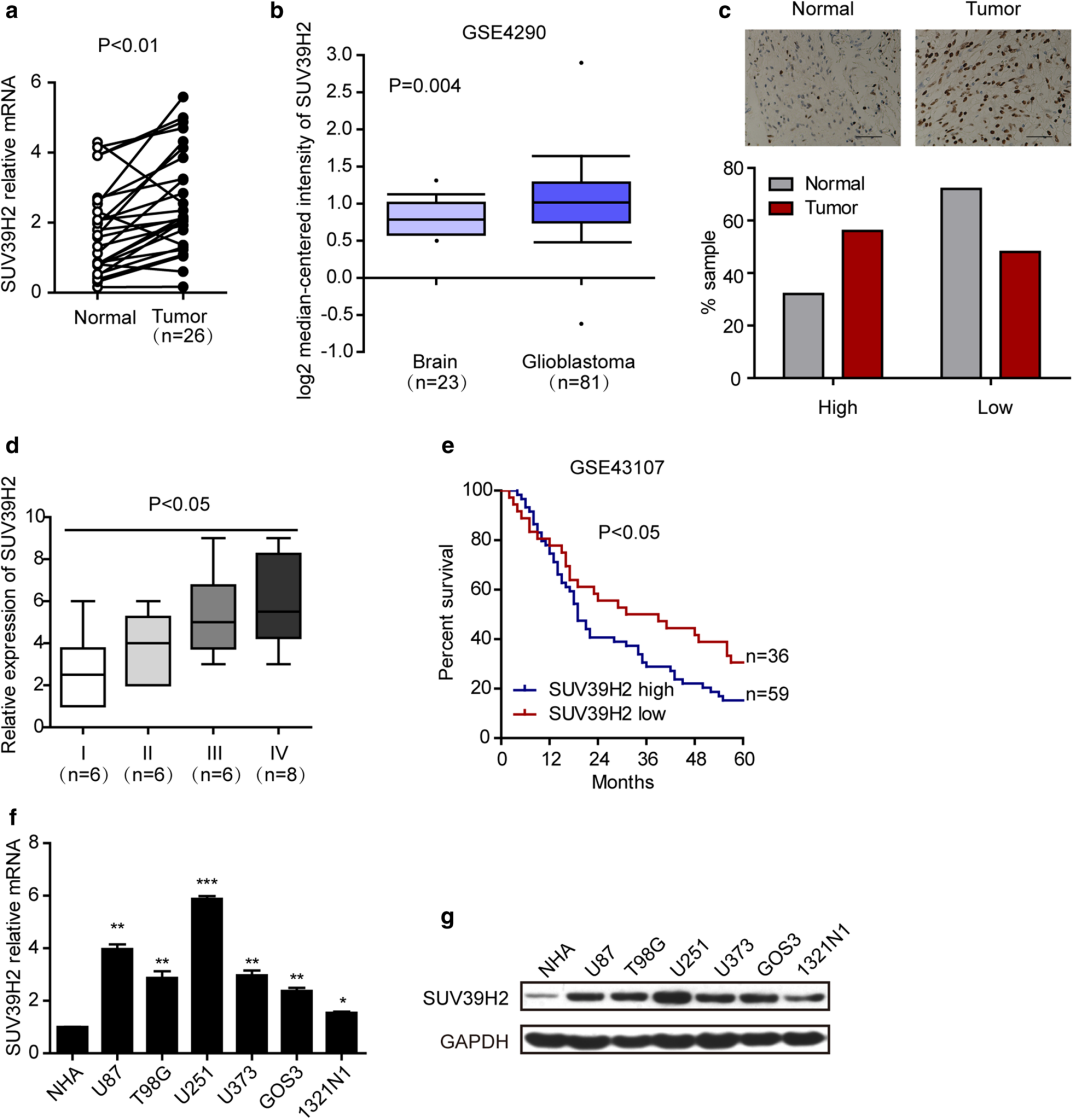
Relevance of SUV39H2 expression in human glioma. **a** The mRNA expression levels of SUV39H2 were assessed by RT-qPCR in 26 tumor tissues and their matched normal tissues (paired t test). **b** In the GSE4290 database, the mRNA expression levels of SUV39H2 were analyzed in glioblastoma and normal tissues (t test). **c** SUV39H2 was assessed in glioma tissues (n = 26) and matched normal tissues (n = 26) by IHC staining. A 10-point quantification scale (1–5, low; 6–10, high) was applied to indicate SUV39H2 staining indexes (χ^2^ test, p < 0.05). **d** A 10-point quantification scale (1–5, low; 6–10, high) was applied to indicate SUV39H2 staining indexes in different grades of glioma tissues (Kruskal–Wallis test). **e** Kaplan–Meier survival curves of patients based on SUV39H2 expression in the GSE43107 database (log-rank test). **f**, **g** The mRNA (**f**) and protein (**g**) expression levels of SUV39H2 in different glioma cell lines (t test). Data are shown as the mean ± SEM. (*p < 0.05, **p < 0.01, ***p < 0.001)

To assess the vital function of SUV39H2 in glioma, we conducted RT-qPCR to examine the mRNA expression of SUV39H2 in glioma tissues and paired peritumoral normal tissues. RT-qPCR analysis revealed that SUV39H2 mRNA was remarkably upregulated in 26 glioma tissues compared to their normal counterparts (Fig. 1a). We further examined SUV39H2 expression in the public Oncomine database. The results showed that SUV39H2 was also higher in glioma tissues than in normal brain tissues in the GSE4290 database (Fig. 1b). Simultaneously, IHC analysis showed that SUV39H2 expression was significantly higher in glioma tissues (Fig. 1c). Correlation studies showed that SUV39H2 expression was positively linked to the glioma grade (Fig. 1d). More importantly, the clinical relevance of SUV39H2 was verified by data mining and analysis of the public database. As represented in Fig. 1e, SUV39H2 expression was associated with survival in 2 separate groups in the GSE43107 database. The group with higher SUV39H2 expression had significantly lower survival rates. In addition, we tested SUV39H2 expression in six glioma cell lines and the NHA cell line, a normal human astrocyte cell line. We also observed that the RNA and protein levels of SUV39H2 in glioma cell lines were higher (Fig. 1f, g). Taken together, these data highlight that SUV39H2 is a potential biomarker for glioma and indicates the causal relationship between SUV39H2 and glioma tumorigenesis.

### SUV39H2 promotes the proliferation of glioma cells in vitro

**Fig. 2:**
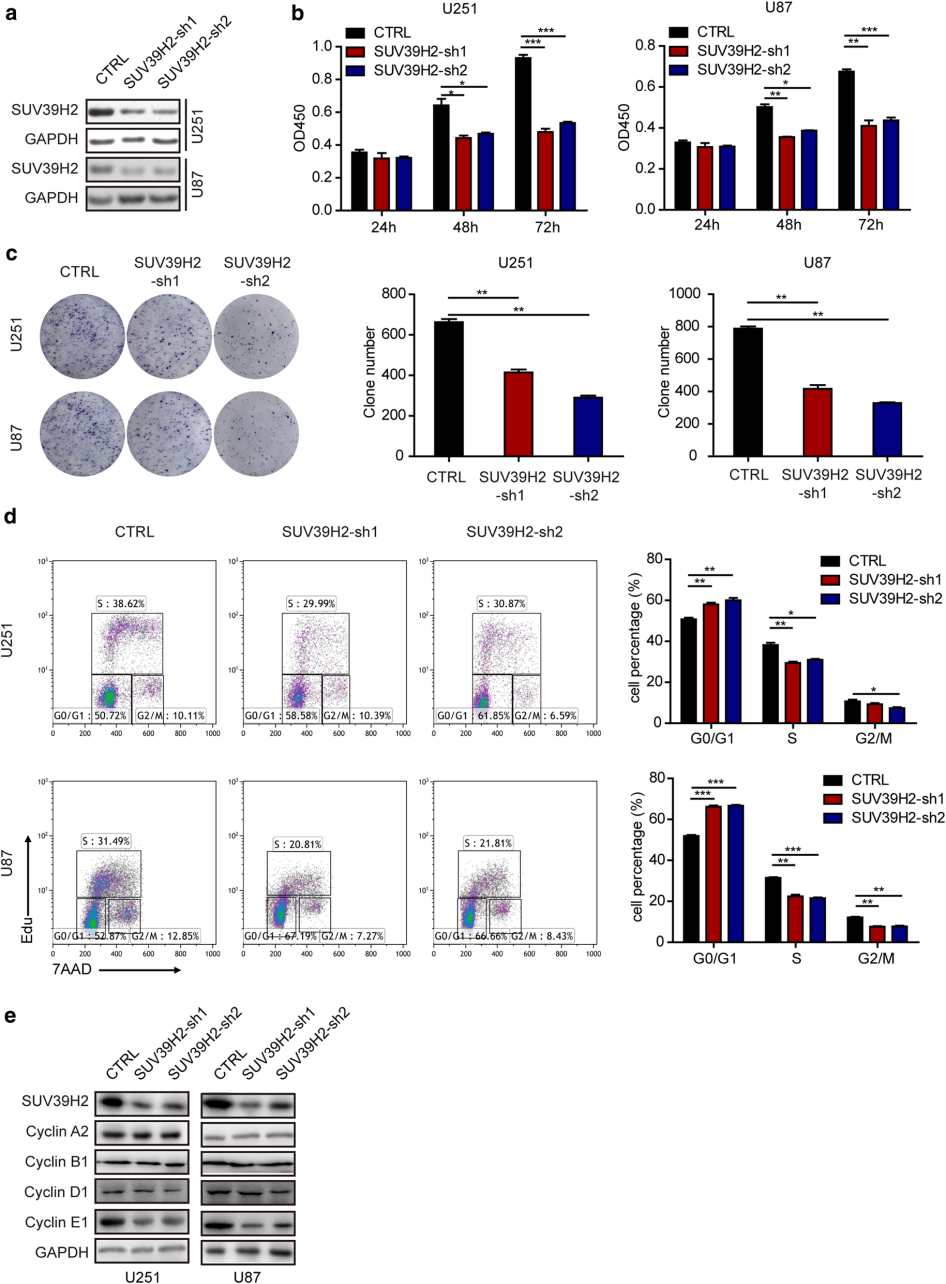
SUV39H2 knockdown inhibits the tumorigenesis of glioma cells in vitro. **a** The expression of the indicated proteins assessed by WB in control (CTRL) and SUV39H2-knockdown (SUV39H2-sh) U251 or U87 cells. **b** Cell viability was assessed by the CCK-8 assay in CTRL and SUV39H2-sh U251 or U87 cells. **c** Representative images and cartograms of the colony formation assay in CTRL and SUV39H2-sh U251 or U87 cells. **d** Flow cytometry was performed to assess the effects of SUV39H2 on the cell cycle in CTRL and SUV39H2-sh U251 or U87 cells. **e** The expression of the indicated proteins assessed by WB in control (CTRL) and SUV39H2-knockdown (SUV39H2-sh) U251 or U87 cells. Data are shown as the mean ± SEM of at least three independent experiments. (*p < 0.05, **p < 0.01, ***p < 0.001, Student’s t-test)

To explore the function of SUV39H2 in glioma cells, SUV39H2 was knocked down in U251 and U87 cell lines by the lentiviral system with 2 shRNA sequences targeting SUV39H2 (SUV39H2-sh1 and SUV39H2-sh2) or a scrambled sequence as a control (CTRL). The knockdown efficiency of SUV39H2 in U251 and U87 cells was confirmed by WB analysis (Fig. 2a and Additional file 2: Figure S1). Using the CCK-8 assay, we observed that reduced SUV39H2 expression inhibited glioma cell growth (Fig. 2b). These results were further confirmed by colony formation assays (Fig. 2c). The results revealed that SUV39H2 had important functions in glioma progression in glioma cell lines. Further, flow cytometric analysis was applied to determine the function of SUV39H2 in the progression of the glioma cell cycle. We observed that reduced SUV39H2 expression inhibited the G1/S phase transition (Fig. 2d). Furthermore, we found that Cyclin E1 was the most relevant cyclin contributing to the cell cycle checkpoint (Fig. 2e). Together, these results demonstrate that SUV39H2 promotes the proliferation of glioma cells by affecting cell cycle progression.

### SUV39H2 promotes the tumorigenesis of glioma cells in vivo

**Fig. 3:**
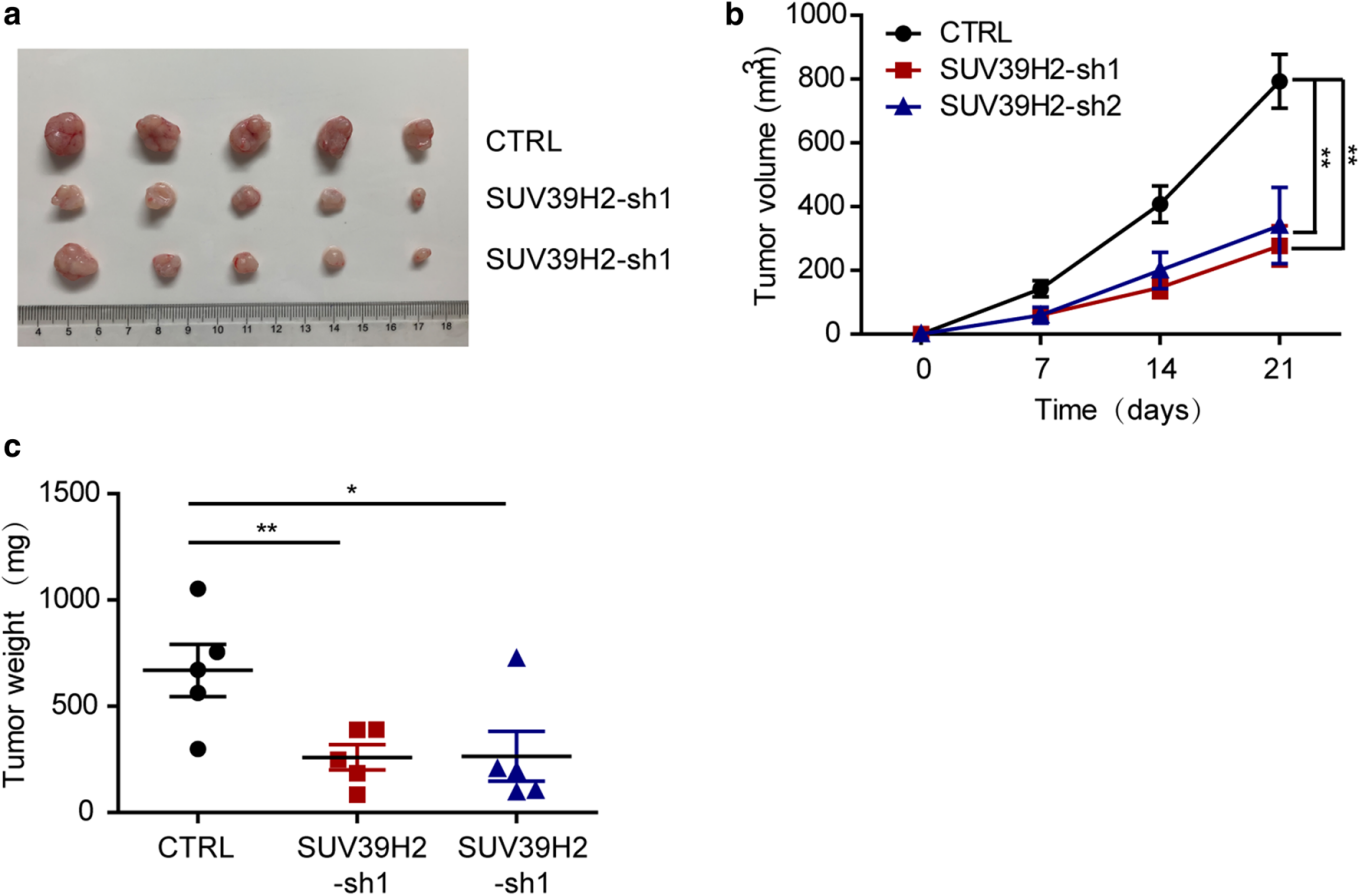
SUV39H2 is essential for glioma cell growth in vivo. **a** Image of tumors from nude mice in the indicated groups (n = 5). **b** The tumor volume was recorded on different days. **c** The weight of the tumor was recorded when the mice were sacrificed on day 21. Data are shown as the mean ± SEM. (*p < 0.05, **p < 0.01, ***p < 0.001, Student’s t-test)

We found that SUV39H2 is necessary for the proliferation of glioma cells in vitro. Next, we aimed to define the role of SUV39H2 in glioma progression in vivo using a xenograft mouse model. The stable SUV39H2-knockdown U251 cell line, which was acquired after being transfected and screened, was injected into nude mice subcutaneously. We recorded tumor volume weekly and measured tumor weight on the 21st day after sacrifice (Fig. 3a–c). The data showed that the weight and size of the gliomas were dramatically reduced when SUV39H2 was knocked down. These results imply that SUV39H2 promotes the tumorigenesis of glioma cells in vivo.

### SUV39H2 sustains the properties of glioma stem cells

**Fig. 4:**
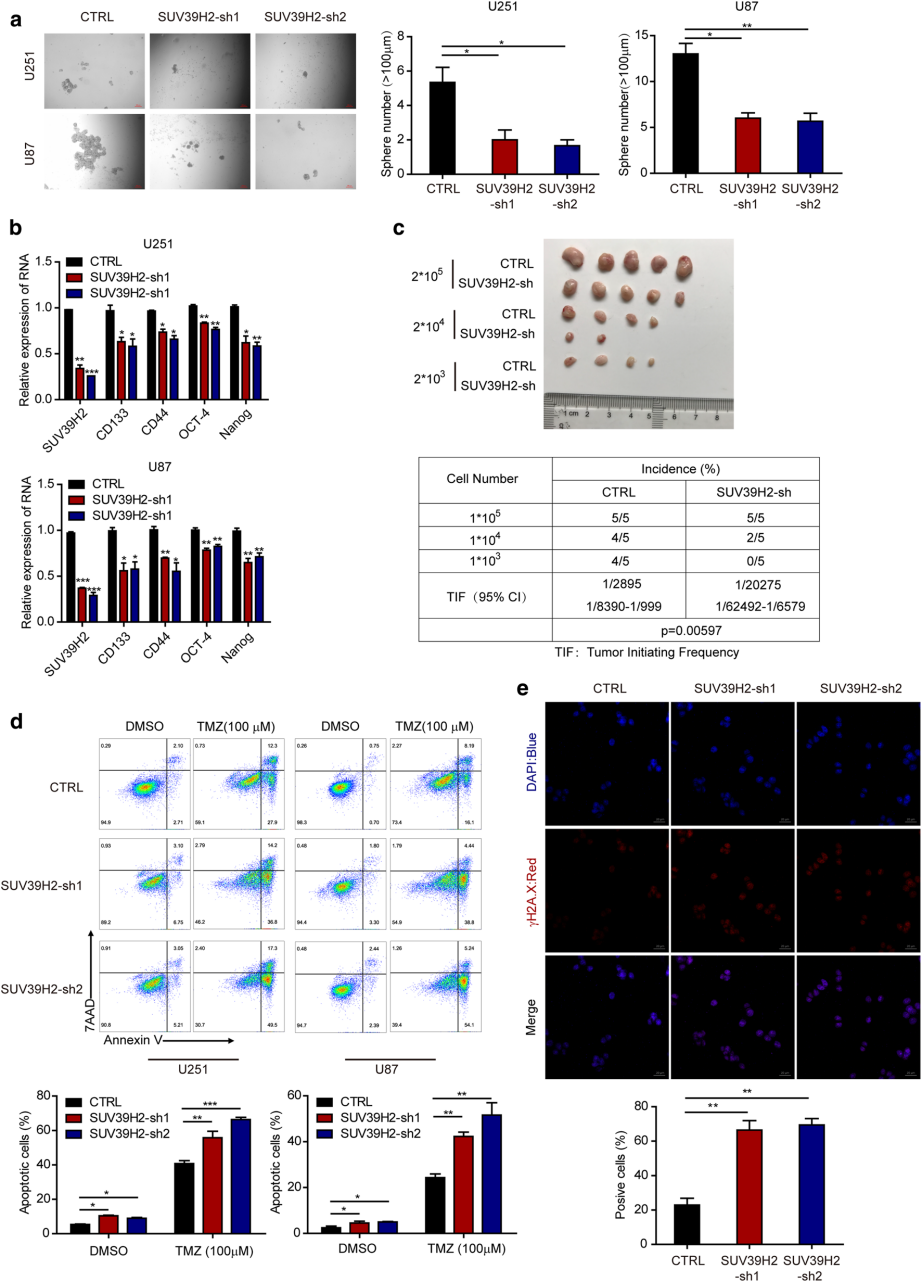
SUV39H2 knockdown inhibits cancer stem cell properties and promotes apoptosis induced by TMZ in glioma cells. **a** Sphere formation capacity of CTRL and SUV39H2-sh U251 or U87 cells (scale bar, 200 μm). **b** mRNA expression of the indicated genes related to stemness was assessed in U251 or U87 cells. **c** In *vivo* tumor initiation assay of CTRL and SUV39H2-sh glioma cells. **d** An annexin-V/7AAD assay was performed to measure cell apoptosis in CTRL and SUV39H2-sh cells in the absence or presence of 100 μM TMZ for 48 h. **e** γH2A.X formation was measured by IF staining in U251 cells treated with 100 μM TMZ for 24 h. Data are shown as the mean ± SEM of at least three independent experiments. (*p < 0.05, **p < 0.01, ***p < 0.001, Student’s t-test)

Glioma stem-like cells (GSCs) are a particular group of cells within the glioma mass. They display the properties of stem cells and have been implicated in tumor recurrence and drug resistance. As GSCs have the capacity of self-renewal, are pluripotent, and induce tumorigenesis [9–12], we next tested whether SUV39H2 could regulate the GSC phenotype. We conducted a sphere formation experiment and discovered that SUV39H2 knockdown decreased the number of glioma tumorspheres (Fig. 4a). These results indicate that SUV39H2 is associated with sphere formation characteristics of GSCs. Moreover, we detected the expression of genes related to stemness (CD133, CD44, OCT-4, and Nanog) by RT-qPCR. The mRNA expression levels of these genes decreased following SUV39H2 knockdown (Fig. 4b). As tumor initiation is one of the critical roles of GSCs, we conducted an in vivo tumor initiation assay to further demonstrate the role of SUV39H2 in sustaining GSC stemness. The results showed that mice implanted with control cells developed tumors with a significantly higher incidence rate compared with mice implanted with SUV39H2-knockdown cells (Fig. 4c). In summary, these results highlight that SUV39H2 sustains GSC characteristics.

### SUV39H2 deficiency enhances TMZ sensitivity in glioma cells

Drug resistance is one of the troublesome problems in clinical glioma treatment. GSCs have been implicated in drug resistance. Thus, we treated SUV39H2-knockdown and control cells with TMZ to investigate the function of SUV39H2 in the chemosensitivity of glioma by flow cytometric analysis. The results showed that SUV39H2 deficiency increased the percentage of apoptotic glioma cells induced by TMZ (Fig. 4d), which indicates that SUV39H2 deficiency makes glioma cells more sensitive to TMZ treatment. A hallmark of TMZ-induced glioma apoptosis is γH2A.X foci formation. As confirmed by immunofluorescence analysis, the knockdown of SUV39H2 resulted in dramatically more γH2A.X-positive cells (Fig. 4e). Together, these data demonstrate that the knockdown of SUV39H2 promotes the chemosensitivity of glioma cells to TMZ treatment.

### SUV39H2 promotes hedgehog signaling by downregulating HHIP in glioma cells

**Fig. 5:**
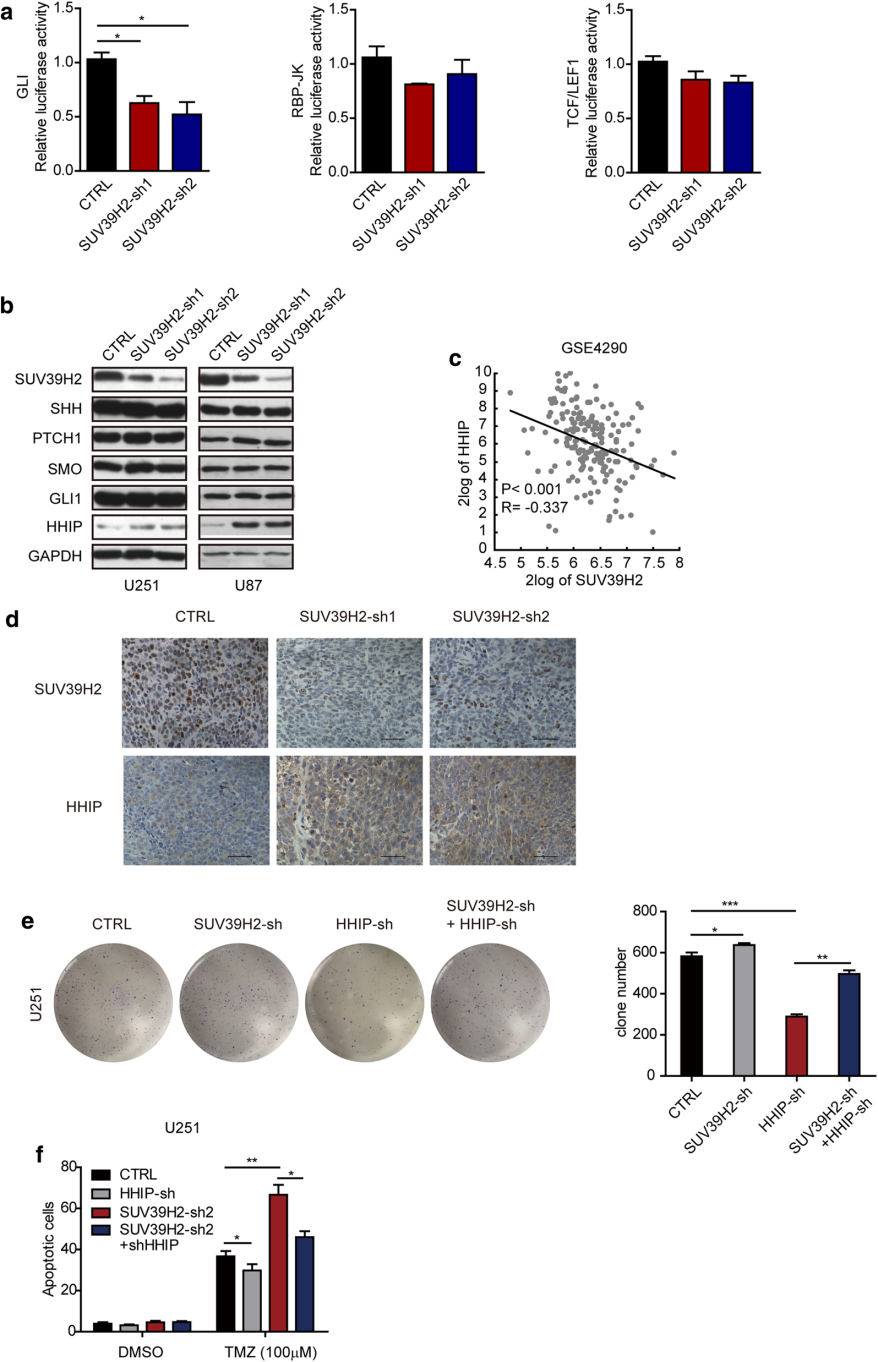
SUV39H2 regulates the properties of cancer stem cells in glioma cells by modulating hedgehog signaling. **a** RBP-Jκ, GLI, and TCF/LEF1 responsive luciferase reporter assays were performed in CTRL and SUV39H2-sh glioma cells. **b** Western blot analysis of the indicated proteins related to Hh signaling in U251 and U87 cells. **c** Correlation between SUV39H2 and HHIP levels in glioma patients (n = 180) from the GSE4290 database (http://r2.amc.nl). **d** Representative images of SUV39H2 and HHIP IHC staining in the indicated xenograft tumors. **e** Representative images and cartograms of the colony formation assay in CTRL, SUV39H2-sh, HHIP-sh, and SUV39H2-sh plus HHIP-sh U251 cells. **f** An annexin-V/7AAD assay was performed to measure cell apoptosis induced by TMZ in CTRL, SUV39H2-sh, HHIP-sh, and SUV39H2-sh plus HHIP-sh U251 cells. Data are shown as the mean ± SEM of at least three independent experiments. (*p < 0.05, **p < 0.01, ***p < 0.001, Student’s t-test)

The Notch, Wnt, and hedgehog (Hh) signaling pathways play important roles in glioma progression. These pathways are important for sustaining the properties of GSCs. To gain insight into the impact of SUV39H2 on the underlying mechanism of glioma, we conducted luciferase reporter assays with hedgehog (GLI), Notch (RBP-JK), and Wnt (TCF/LEF1) luciferase reporters. We found that the activity of only the GLI luciferase reporter was significantly reduced in SUV39H2-knockout glioma cells relative to normal cells (Fig. 5a). Furthermore, we examined the protein levels of key components (SHH, PTCH1, GLI 1, SMO, and HHIP) of hedgehog signaling. SUV39H2 knockdown resulted in increased HHIP expression but had no impact on other components (Fig. 5b). Moreover, the reverse correlation between SUV39H2 and HHIP in glioma cells was further confirmed by analyzing the GSE4290 database (Fig. 5c). To further confirm that HHIP was regulated by SUV39H2 in glioma, we examined the expression of SUV39H2 and HHIP in xenograft tumors using immunohistochemistry (IHC) staining. High HHIP expression was observed in SUV39H2-sh tumors (Fig. 5d). Thus, SUV39H2 negatively regulates HHIP expression. Furthermore, to determine whether SUV39H2 regulates glioma progression in an HHIP-dependent way, we transfected shSUV39H2 or the combination of shSUV39H2 and shHHIP into U251 cells. The results showed that the suppression of cell growth induced by the knockdown of SUV39H2 was partially reversed by the knockdown of HHIP in U251 cells (Fig. 5e). Similarly, TMZ sensitivity caused by SUV39H2 deficiency was also partially reversed by the knockdown of HHIP in glioma cells (Fig. 5f). In summary, these data demonstrate that SUV39H2 regulates tumor growth and TMZ sensitivity in an HHIP-dependent manner in glioma cells.

## Discussion

Malignant gliomas are incurable tumors that represent the majority of all primary brain tumors [1]. Even after the gold standard treatment, the median survival time of glioblastoma patients is still less than 15 months [7]. The poor prognosis is due to tumor recurrence and drug resistance. Thus, finding the underlying mechanisms of glioma development and recurrence is urgently needed.

Many changes in genetic and epigenetic mechanisms in glioma have been reported [17]. Because of their reversibility, epigenetic changes are more promising therapeutic targets than genetic changes [24, 25]. Simultaneously, histone modifications have important roles in the tumorigenesis of glioma [26–28]. Moreover, H3K9me3 plays a key role in the differentiation of glial cells and affects the survival of patients with high-grade astrocytomas [27]. SUV39H1, an H3K9 methyltransferase, has been implicated in the progression of glioma [27, 29]. However, SUV39H2, a homologous enzyme of SUV39H1, is upregulated in many cancers, but it is still unknown whether it is upregulated in glioma. Here, we primarily elucidated that SUV39H2 is upregulated in human glioma tissues and glioma cell lines. There is a positive correlation between SUV39H2 expression and the grade of glioma progression. Additionally, SUV39H2 is essential in regulating the growth of glioma cells in vitro and in vivo. Therefore, SUV39H2 may be a new marker to predict the progression of glioma.

GSCs have the capacity to self-renew, are pluripotent, and induce tumorigenesis [9–12]. Additionally, repressive histone methylation redistribution is observed in GSCs [30]. We conducted a series of experiments to discover the relation between GSCs and SUV39H2. In our study, we found that SUV39H2 was associated with the characteristics of GSCs and that the knockdown of SUV39H2 repressed the expression of GSC-related genes. GSCs have been implicated in tumor recurrence and drug resistance. We delightedly found that the knockdown of SUV39H2 contributed to TMZ sensitivity in glioma cells. Thus, these data indicate that SUV39H2 may be a promising target to cure drug-resistant glioma.

The hedgehog signaling pathway plays pivotal roles in glioma progression [13–15]. The low expression or inactivation of HHIP has been reported in many tumors, including glioma [31]. The low expression of HHIP is epigenetically regulated, as its promoter is overly methylated in glioma [32]. However, one study showed that low HHIP expression was caused by chromatin remodeling in the gastrointestinal tract [33]. These results imply that HHIP repression in glioma may be regulated by repressive histone methylation. In our study, we found that SUV39H2 regulated the activity of the GLI1 reporter, and there was a reverse correlation between SUV39H2 and HHIP expression in glioma. Next, we demonstrated that SUV39H2 regulated cell growth and chemosensitivity in glioma by regulating hedgehog signaling.

SUV39H2 and its homologous enzyme SUV39H1 have functional redundancy in embryonic development [34]. However, they possess different functions in cancers [35]. Despite its tumor suppressor role in rhabdomyosarcoma [36], SUV39H1 promotes tumorigenesis in glioma [27, 29]. In the GSE4290 database, we also found that SUV39H1 has a reverse correlation with HHIP, which indicates that SUV39H2 and SUV39H1 may have mutual compensatory effects on tumor growth and chemosensitivity in glioma. The mutual compensatory effects of SUV39H2 and SUV39H1 in glioma need to be further elucidated. It has been reported that the histone methyltransferases EZH2, which methylates H3K27, modifies the nonhistone protein by directly binding to STAT3 in glioblastoma. Thus, whether SUV39H2 regulates HHIP in this manner or by histone methylation needs to be further explored.

In summary, we primarily revealed the effect of SUV39H2 on cell growth and chemosensitivity to TMZ in glioma. We found that SUV39H2 regulated Hh signaling via HHIP. These findings may provide a new treatment strategy to inhibit glioma cell growth and enhance chemosensitivity by combining SUV39H2 inhibitors with chemotherapy. In addition, SUV39H2 could be a diagnostic biomarker of glioma. Thus, we elucidated a new marker and strategies for clinical glioma therapeutic regimens.

## Conclusions

In summary, we found that SUV39H2 is highly expressed in glioma cells and glioma tissues. SUV39H2 is positively correlated with the progression (grade) of glioma and patient survival rates. SUV39H2 knockdown represses cell growth and promotes cell chemosensitivity in glioma cells by upregulating HHIP expression. Together, SUV39H2 can be a new biomarker and a new strategy for clinical glioma therapeutic regimens.

## Supplementary information

**Additional file 1: Table S1.** Sequences of shRNA. **Table S2.** Sequences of RT-PCR primers. **Table S3.** Antibodies used in Immunoblotting, Immunohistochemistry and Immunofluorescence experiments.

**Additional file 2: Figure S1.** The SUV39H2 shRNA have no non-specific effect on SUV39H1.

## Data Availability

Address the corresponding author.

## Abbreviations

CCK-8: Cell Counting Kit-8
DAKO: diaminobenzidine
GSCs: glioma stem-like cells
GBM: glioblastoma
HHIP: hedgehog interacting protein
HRP: horseradish peroxidase
Hh: hedgehog
H3K9: lysine 9 of histone 3
IHC: immunohistochemistry
IF: immunofluorescence
PVDF: polyvinylidene difluoride
RT-qPCR: real-time reverse transcription polymerase chain reaction
SUV39H2: suppressor of variegation 3–9 homolog 2
SDS-PAGE: sodium dodecyl sulfate–polyacrylamide gel electrophoresis
TMZ: temozolomide
WHO: World Health Organization
WB: western blot
