# Supplementary material for: Histone methyltransferase SUV39H2 regulates cell growth and chemosensitivity in glioma via regulation of hedgehog signaling
Source: Cancer Cell Int. 2019 Oct 16;19:269. doi: 10.1186/s12935-019-0982-z (PMC6794832; doi:10.1186/s12935-019-0982-z)
Supplement: Supplementary file 1 — Additional file 1: Table S1. Sequences of shRNA. Table S2. Sequences of RT-PCR primers. Table S3. Antibodies used in Immunoblotting, Immunohistochemistry and Immunofluorescence experiments. [file 12935_2019_982_MOESM1_ESM.doc]

Table S1 Sequences of shRNA

| SUV39H2-sh1 | CCAAATCTTCAGGTGTTCAAT |
| --- | --- |
| SUV39H2-sh2 | CTCTAATGACAAGCATAATTA |
| HHIP-sh | GCAACGTGCCTTATTCCATAC |

Table S2 Sequences of RT-PCR primers

| GAPDH-F | TGACGTGGACATCCGCAAAG |
| --- | --- |
| GAPDH-R | CTGGAAGGTGGACAGCGAGG |
| SUV39H2-F | ATTGATAACCTCGATACTCGTCTT |
| SUV39H2-R | TCTCCAGAACCTTTCATTTGATAA |
| OCT-4-F | CTGGGTTGATCCTCGGACCT |
| OCT-4-R | CCATCGGAGTTGCTCTCCA |
| CD44-F | TCCAACACCTCCCAGTATGACA |
| CD44-R | GGCAGGTCTGTGACTGATGTACA |
| CD133-F | CCTACAGGGCGTGGTGGT |
| CD133-R | GTCCGGCCTCATCGAGAC |
| Nanog-F | TTTGTGGGCCTGAAGAAAACT |
| Nanog-R | AGGGCTGTCCTGAATAAGCAG |

Table S3 Antibodies used in Immunoblotting, Immunohistochemistry and Immunofluorescence experiments

| Antibodies | Catalog |
| --- | --- |
| SUV39H2 | ab240313/ab190870,Abcam |
| SHH | ab53281, Abcam |
| GAPDH | ab8245, Abcam |
| HHIP | ab39208, Abcam |
| SMO | ab236465, Abcam |
| GLI1 | ab49314, Abcam |
| PTCH1 | ab53715, Abcam |
| Alexa Fluor-488 anti-rabbit IgG | A21206 Life Technologies |
| γH2A.X | Ab2893, Abcam |
| SUV39H1 | Ab38637, Abcam |
| Cyclin A2 | 91500S, CST |
| Cyclin E1 | 20808S, CST |
| Cyclin B1 | 4138S, CST |
| Cyclin D1 | 2922S, CST |
|  |  |
