# Supplementary figures and images for: Histone methyltransferase SUV39H2 regulates cell growth and chemosensitivity in glioma via regulation of hedgehog signaling
Source: Cancer Cell Int. 2019 Oct 16;19:269. doi: 10.1186/s12935-019-0982-z (PMC6794832; doi:10.1186/s12935-019-0982-z)

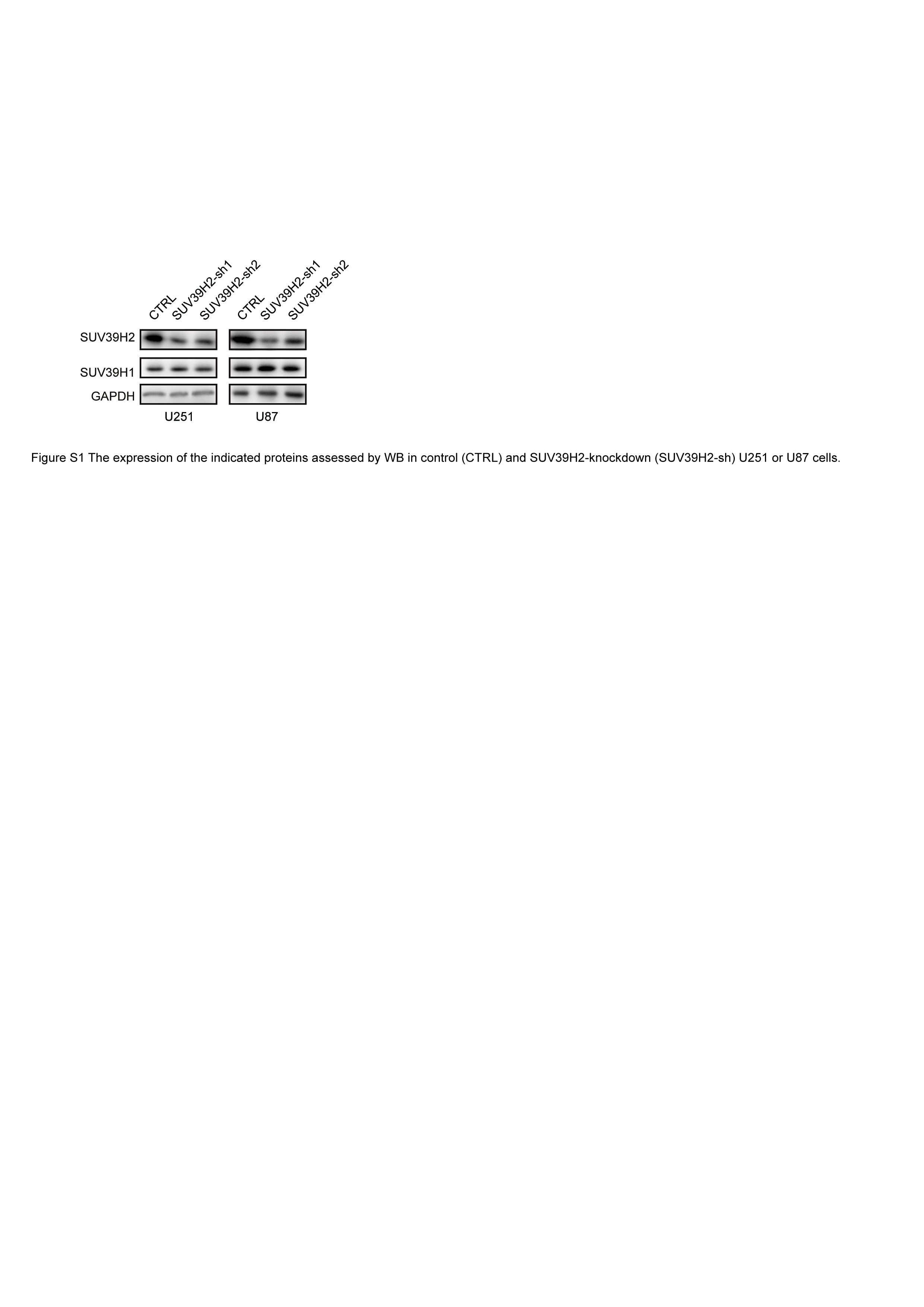

Supplement: Supplementary file 2 — Additional file 2: Figure S1. The SUV39H2 shRNA have no non-specific effect on SUV39H1. [file 12935_2019_982_MOESM2_ESM.tif]
